# Performance of a Knowledge-Based Model for Optimization of Volumetric Modulated Arc Therapy Plans for Single and Bilateral Breast Irradiation

**DOI:** 10.1371/journal.pone.0145137

**Published:** 2015-12-21

**Authors:** Antonella Fogliata, Giorgia Nicolini, Celine Bourgier, Alessandro Clivio, Fiorenza De Rose, Pascal Fenoglietto, Francesca Lobefalo, Pietro Mancosu, Stefano Tomatis, Eugenio Vanetti, Marta Scorsetti, Luca Cozzi

**Affiliations:** 1 Radiotherapy and Radiosurgery Department, Humanitas Clinical and Research Center, Milan-Rozzano, Italy; 2 Oncology Institute of Southern Switzerland, Bellinzona, Switzerland; 3 Radiotherapy Department, ICM-Val d’Aurelle, Montpellier, France; University Medical Centre Utrecht, NETHERLANDS

## Abstract

**Purpose:**

To evaluate the performance of a model-based optimisation process for volumetric modulated arc therapy, VMAT, applied to whole breast irradiation.

**Methods and Materials:**

A set of 150 VMAT dose plans with simultaneous integrated boost were selected to train a model for the prediction of dose-volume constraints. The dosimetric validation was done on different groups of patients from three institutes for single (50 cases) and bilateral breast (20 cases).

**Results:**

Quantitative improvements were observed between the model-based and the reference plans, particularly for heart dose. Of 460 analysed dose-volume objectives, 13% of the clinical plans failed to meet the constraints while the respective model-based plans succeeded. Only in 5 cases did the reference plans pass while the respective model-based failed the criteria. For the bilateral breast analysis, the model-based plans resulted in superior or equivalent dose distributions to the reference plans in 96% of the cases.

**Conclusions:**

Plans optimised using a knowledge-based model to determine the dose-volume constraints showed dosimetric improvements when compared to earlier approved clinical plans. The model was applicable to patients from different centres for both single and bilateral breast irradiation. The data suggests that the dose-volume constraint optimisation can be effectively automated with the new engine and could encourage its application to clinical practice.

## Introduction

Early pre-clinical and clinical experiments [1–7] demonstrated the feasibility of automated and a personalised definition of appropriate dose-volume optimization constraints starting from the modelling of historical planning data. With the use of this so-called knowledge-based planning, evidence has accumulated on the improved plan quality, reduced inter-clinician variability, and the possibility to transfer the planning expertise from more experienced centres to less experienced institutions.

A commercial engine aiming to perform the above tasks was recently developed and released by Varian Medical Systems (Palo Alto, USA), the RapidPlan system. It takes the geometrical features of the patients into account, and correlates these to previously achieved dosimetry, in order to generate appropriate location of the dose-volume objectives for the optimization phase. Early pre-clinical validation studies have been published [8–10] investigating its role for the planning of liver, prostate, lung and head and neck, using volumetric modulated arc therapy (VMAT). In all these studies, the primary focus was the appraisal of the quality of the models built from relatively limited sets of patients, and the determination of efficient methods for the model validation.

The present study aimed to investigate the quality of a model based automated optimisation for VMAT plans of whole breast irradiation. VMAT, in its RapidArc form was appraised for breast treatment in a number of pre-clinical and clinical studies [11–20]. These investigations assessed the feasibility of the RapidArc treatments, providing clinically good dose distributions (in terms of target coverage and organs at risk (OAR) sparing). Clinical results also showed good compliance from patients and minimal incidence of acute toxicity [16]. The choice of the breast as a site for knowledge-based planning investigation is related to the difficulties in planning these cases with VMAT technology, and to the associated need for delineating additional structures to improve conformity and decrease the dose to the surrounding structures, such as lungs and heart. This leads to a time consuming planning procedure, often hampering optimal plan quality being obtained. Moreover, as the models used in the automated optimisation process are generated by a multitude of different patients and geometries, this variability could possibly also lead to good results for patients presenting with very complex anatomies.

The goals of this study were: i) development of an automated dose-volume histogram objectives prediction model for whole breast irradiation with simultaneous integrated boost (SIB); ii) *in-silico* validation of the model on groups of patients from different institutions to appraise the robustness of the engine to different contouring and planning aims; iii) evaluation of the generalisation power of the model to the case of bilateral breast irradiation with SIB.

## Material and Methods

A new knowledge-based optimisation engine, named RapidPlan, was introduced in the Eclipse treatment planning system (Varian Medical Systems, Palo Alto, USA) from its release 13.5 [21–22]. First studies on pre-clinical validation have recently been published [8–10] and include some details on its implementation in Eclipse. The aspects of RapidPlan investigated in the present study are: i) the model building and training (Dose Volume Histogram (DVH) Estimation Model Configuration); ii) the automated model based dose-volume objectives prediction tool (DVH Estimation).

**The DVH Estimation Model Configuration:** In order to configure a model, a set of geometric and dosimetric information are extracted from a group of selected available treatment plans. A combination of Principal Component Analysis and regression techniques, PCA-regression [23], extracts the features that are used in the DVH estimation phase [21–22]

**The DVH Estimation:** in this phase, for any new patient, the DVH ranges for the structures defined in the model are predicted. These are specific for the new patient anatomy derived from the features extracted in the model training. These ranges are used to automatically create the optimization objectives, as line objectives just below the predicted DVH bands.

### Breast Model Configuration

One hundred andfifty patients (75 left-sided, 75 right-sided breasts), all treated for whole breast with simultaneous integrated boost with RapidArc were selected for training the DVH estimation model. All plans were approved for clinical use and details of the treatment protocol can be found in [16]. All patient data were anonymised. No ethical committee approval was needed for this kind of investigations. All the plans selected for model training were checked for their quality prior to their inclusion in the model. Whenever a single structure of a specific patient was suggested as a potential outlier by the metrics during the model configuration phase, the case was carefully re-checked to look for possible odd anatomic or dosimetric features. They were found to be not influential for the model quality (anatomically and dosimetrically reliable plans) and therefore not considered as real outliers. For this reason those cases were not excluded from the training dataset. Normal distribution of data was assessed and confirmed.

The boost clinical target volume (CTV) was defined as 1 cm around the surgical clips placed in the lumpectomy area, while the breast CTV was defined as the entire mammary gland. The planning target volumes (PTV) were defined by adding an isotropic margin of 5mm to the corresponding CTVs (3mm for organ movement due to respiration, and 3mm for patient set-up, summed in quadrature); the breast PTV was cropped 4–5mm inside the body outline to exclude the skin and also cropped to exclude the ribs and the lung parenchyma. A total dose of 48.0Gy in 15 fractions was prescribed to the boost volume (PTV_boost), and simultaneously 40.5Gy to the whole breast PTV. PTV_breast volumes ranged from 116 to 2062 cm^3^ (mean 648 cm^3^), PTV_boost volumes ranged from 11 to 269 cm^3^ (mean 68 cm^3^). The OARs included in the training were: ipsi- and contra-lateral lungs, contra-lateral breast, heart (defined as ipsi- or contra-lateral depending on the laterality of the tumour), spinal cord, oesophagus and trachea. The model was configured to generate line-type and mean-dose objectives for all involved OARs with optimisation priorities and line-type objectives generated by the system. Constraints and priorities for the PTVs were manually set to predefined values. Table 1 summarises the model objectives.

**Table 1:** Dose-volume constraints as defined in the DVH constraints prediction model.

Structure	Objective type	Relative volume (%)	Dose (% of prescription or absolute dose in Gy)	Priority
PTV_boost	Upper	0	101%	100
	Upper	50	100%	100
	Lower	100	99%	100
	Lower	50	100%	100
PTV_breast	Upper	10	103%	100
	Upper	50	100%	100
	Lower	100	99%	100
	Lower	50	100%	100
Ipsilateral lung	Mean		8.0 Gy	Generated
	Line	Generated	Generated	Generated
Contralateral lung	Mean		3.0 Gy	Generated
	Line	Generated	Generated	Generated
Ipsilateral heart	Mean		4.0 Gy	Generated
	Line	Generated	Generated	Generated
Contralateral heart	Mean		3.0 Gy	Generated
	Line	Generated	Generated	Generated
Contralateral breast	Mean		3.0 Gy	Generated
	Line	Generated	Generated	Generated
Spinal cord	Line	Generated	Generated	Generated
Esophagus	Line	Generated	Generated	Generated
Trachea	Line	Generated	Generated	Generated

### Breast Model Validation

Fifty patients (29 left-sided, 21 right-sided breasts), not used for training the model, were selected to validate the model: 25 (12 left, 13 right) from the same institute (clinic A) who provided the training patients, and 15 (7 left, 8 right) and 10 (all left) from two additional institutes (clinics B and C). All clinical plans were approved for use (reference plans in the following), and re-optimized with RapidPlan and the above detailed model. The patients from clinic B and C were characterised by a different contouring strategy for targets: the breast PTV was allowed to extend to the body surface (clinic B only) and to include part of the ribs and lung if reached by the CTV to PTV expansion. The dose prescription for patients from clinic B was the same as for clinic A, while clinic C prescribed 63.22Gy in 29 fractions to the PTV_boost, simultaneously to 52.2Gy to the PTV_breast.

The Acuros-XB dose calculation algorithm was adopted with a dose resolution of 2.5mm. RapidArc plans were optimised for 6MV photon beams with generally two partial arcs and collimator angle of 20–30° and the isocenter placed in the center of the breast, for clinics A and B. Additional partial arcs were added in some more challenging cases, always within the limits of anterior-oblique to posterior incidence. Clinic C used a beam arrangement with 4 short arcs and the isocenter placed in the lung, using half beams. Field size was adjusted to cover the target volume in Y and to not exceed 15cm in the X direction. All plans were re-normalised to the mean dose to PTV_boost. DVH analysis on a number of dose-volume objectives was performed to compare the quality of the model-based optimised plans versus the reference plans. Target coverage for PTV_boost aimed to achieve D_1%_<107% and V_95%_>98% with a dose homogeneity expressed by a standard deviation <5%. For PTV_breast, the required coverage was expressed by D_1%_ less than or equal to the dose prescribed to PTV_boost, and D_95%_>95%. The following clinical objectives were used for the plan assessment (derived from [16] and consistent with the criteria used in all three clinics). Ipsilateral lung: V_20Gy_<10% and D_mean_<10Gy. Contralateral breast: D_mean_<3Gy; heart: V_18Gy_<5% and D_mean_<5Gy; contra-lateral lung, spinal cord, oesophagus and trachea: minimisation of the dose as much as possible.

A second validation study was performed by applying the model developed for single breast irradiation to patients requiring bilateral breast treatment. Clinics B and C provided 20 patients (10 each) with dose prescriptions of 64.4Gy and 50.4Gy in 28 fractions to PTV_boost and PTV_breast, respectively (clinic B), and 63.22 Gy and 52.2Gy in 29 fractions to PTV_boost and PTV_breast (clinic C). The laterality of the organs (lungs and heart) was imposed as ipsi-lateral for both sides, and no contralateral breast was matched.

To further appraise the RapidPlan data in comparison with the reference plans, for each patient, and all PTV and OAR objectives, a pass-fail analysis was performed. Data were grouped in 4 classes: failed-passed cases (reference-failed and RapidPlan-passed), passed-failed, failed-failed, and passed-passed.

The plan comparison between clinical and RapidPlan results was statistically evaluated with two-tail paired t-Student tests, to assess differences as significant when p<0.05, and highly significant for p<0.01.

## Results

The model training statistics given by the system showed an acceptable model fit with, among the other parameters, an average chi-square (Pearson) for the regression model parameters of 1.03±0.02.

**Fig 1:**
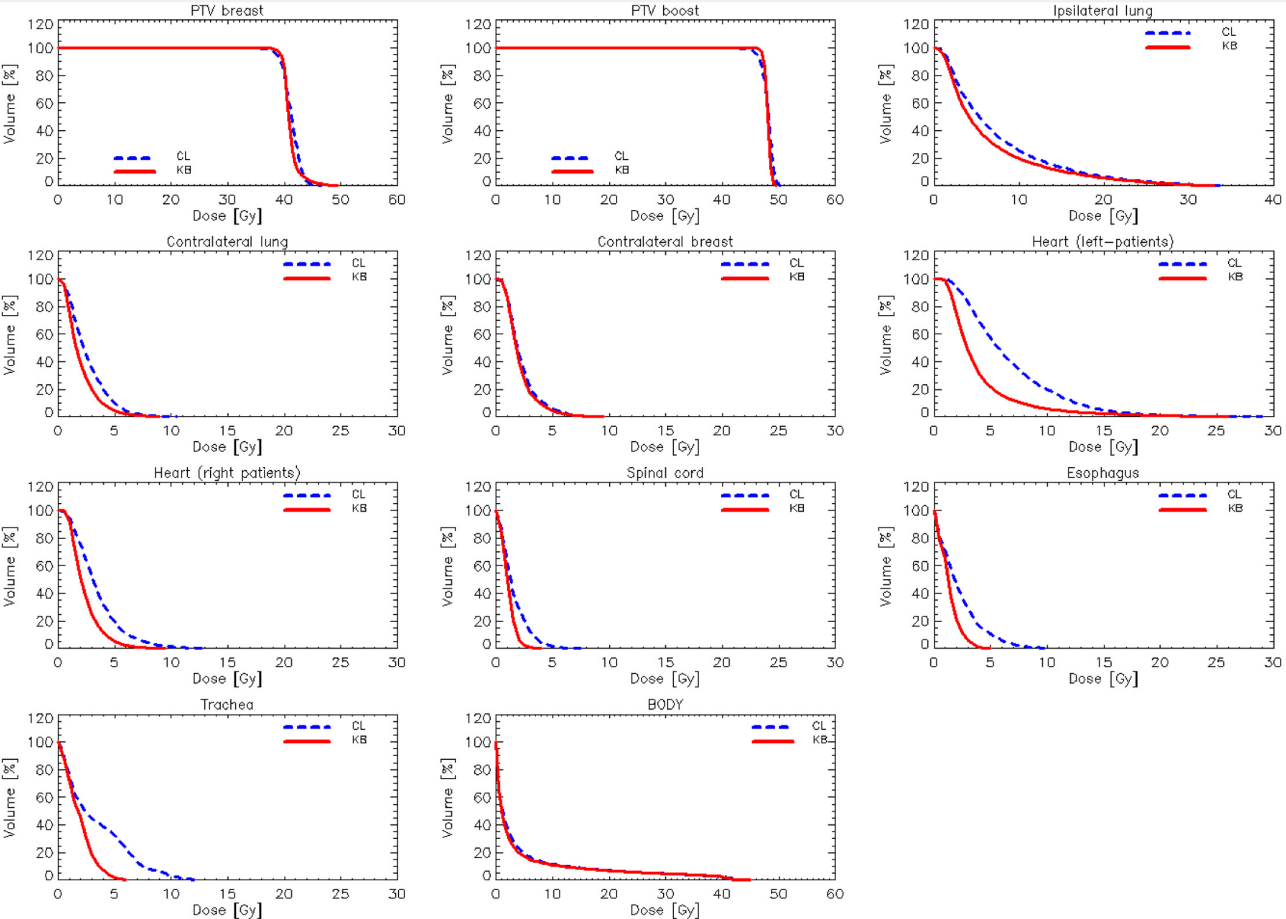
Average DVH for target volumes and organs at risk for the validation experiment for the unilateral breast for clinic A (25 cases). The reference lines (CL) are for the original plans manually optimised, while the RapidPlan lines (KB) are for the model-based optimisation.

**Fig 2:**
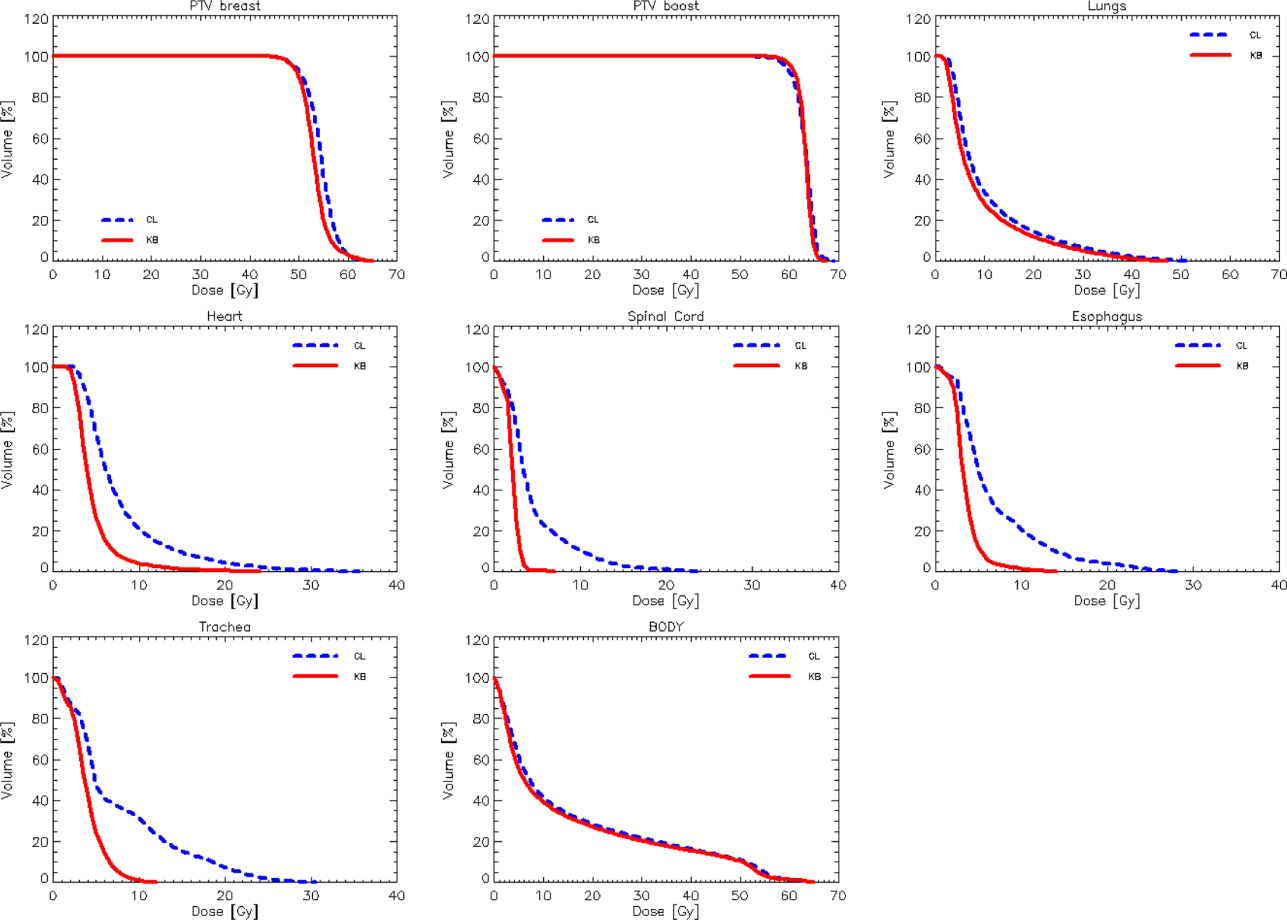
Average DVH for target volumes and organs at risk for the validation experiment on bilateral breast for the clinic B (10 cases). The reference lines (CL) are for the original plans manually optimised, while the RapidPlan lines (KB) are for the model-based optimisation.

Fig 1 shows the DVHs for the target volumes and the various OARs averaged over the 25 test cases from clinic A. Similar graphs were obtained for the data from clinics B and C. Fig 2 shows the average DVH for the target volumes and the various OARs for the 10 test cases for bilateral breast irradiation from clinic B. Table 2 presents the summary of the quantitative analysis of the DVH for the entire group of 50 single breast validation cases divided per clinic; p values are reported only when significant. Table 3 presents the same summary for the bilateral breast validation study.

**Table 2:** Summary of the DVH analysis for the reference and the RapidPlan plans. PTV data are normalised to the respective prescription doses, different for each clinic.

		Clinic-A		Clinic-B		Clinic-C	
	Objective	Reference	RapidPlan	Reference	RapidPlan	Reference	RapidPlan
**PTV boost**							
Mean [%]	100%	100.0±0.0	100.0±0.0	100.0±0.0	100.0±0.0	100.0±0.0	100.0±0.0
D_1%_ [%]	<107%	103.2±0.8	102.1±0.4	103.9±1.1	103.8±1.1	104.6±1.5	103.9±1.0
V_95%_ [%]	>98%	96.2±1.2	99.9±0.1 (**)	95.9±2.9	96.3±1.2	94.4±3.2	96.0±3.2 (*)
St. Dev. [%]	<5%	2.0±0.5	1.0±0.2 (*)	2.2±1.1	2.0±0.7	2.7±0.3	2.3±0.3
**PTV breast**							
Mean [%]	100%	101.9±1.4	101.4±0.5	102.2±2.0	102.4±1.9	103.5±1.9	101.0±1.0 (*)
D_1%_ [%]	<PTV boost	109.7±1.9	115.9±2.4	119.6±1.6	119.3±1.4	113.0±3.4	111.4±4.4
D_95%_ [%]	>95%	95.8±1.6	97.4±0.8 (*)	94.5±3.6	95.0±3.1	94.7±3.3	94.0±2.6
**Ipsilateral lung**							
Mean [Gy]	<10Gy	7.6±0.8	6.5±0.6 (*)	10.3±2.3	9.1±1.8 (*)	7.6±1.8	7.5±1.2
V_20Gy_ [%]	<10%	6.7±2.6	5.8±2.1 (*)	15.1±3.7	13.1±3.7	10.5±4.1	11.1±3.1
**Contralateral lung**							
Mean [Gy]	Minimise	2.7±0.9	2.0±0.5 (*)	3.2±1.2	2.8±1.0 (*)	3.7±0.8	3.2±0.8 (*)
V_20Gy_ [%]	Minimise	0.0±0.0	0.0±0.0	0.1±0.1	0.1±0.1	0.1±0.1	0.1±0.1
**Heart (left-patients)**							
Mean [Gy]	<5Gy	6.8±1.8	4.0±0.4 (*)	5.1±1.0	4.4±0.5 (*)	6.6±1.7	4.9±0.8 (**)
**Heart (right-patients)**							
Mean [Gy]	minimise	3.5±1.2	2.4±0.6 (*)	4.9±2.1	3.6±1.2 (*)	-	-
**Contralateral breast**							
Mean	<3Gy	2.3±0.5	2.1±0.4	3.7±1.1	3.1±0.8 (*)	4.2±0.6	3.3±0.4 (**)
D_1%_ [Gy]	Minimise	6.8±1.3	6.7±1.6	10.6±3.9	9.5±4.4	12.9±3.1	11.4±2.8 (*)
**Spinal Cord**							
D_1%_ [Gy]	Minimise	4.3±1.7	2.5±0.6 (**)	4.0±1.8	3.3±2.8 (*)	3.3±1.8	2.1±0.7 (**)
**Esophagus**							
D_1%_ [Gy]	Minimise	6.8±2.4	1.3±0.5 (**)	6.0±2.8	3.8±2.1 (**)	7.9±3.1	4.4±2.3 (**)
**Trachea**							
D_1%_ [Gy]	Minimise	7.3±3.1	1.9±0.8 (**)	6.6±3.7	5.2±4.5	9.3±5.3	6.4±4.5 (**)

(*): p<0.05

(**): p<0.01; D_x%_: dose receive by at least X% of the volume; V_x%(Gy)_ volume receiving at least C%(Gy) of the dose.

**Table 3:** Summary of the DVH analysis for the reference and the RapidPlan plans for the bilateral breast cases. PTV data are normalised to the respective prescription doses, different for each clinic.

		Clinic-B		Clinic-C	
	Objective	Reference	RapidPlan	Reference	RapidPlan
**PTV boost**					
Mean [%]	100%	100.0±0.0	100.0±0.0	100.0±0.0	100.0±0.0
D_1%_ [%]	<107%	105.1±1.0	103.7±1.2 (*)	105.1±1.4	104.4±1.0 (*)
V_95%_ [%]	>95%	92.2±2.7	96.9±2.4 (**)	92.5±5.4	96.1±2.4 (**)
St. Dev. [%]	<5%	3.1±0.2	2.1±0.2 (*)	3.1±0.4	2.3±0.3 (*)
**PTB breast**					
Mean [%]	100%	102.7±1.7	102.6±0.9	104.3±1.6	102.0±1.0
D_1%_ [%]	<PTV boost	120.9±3.2	124.4±2.7 (*)	117.0±2.8	119.1±2.8
D_95%_ [%]	>95%	94.6±2.0	95.0±1.6	94.4±1.4	93.5±1.0
**Lungs**					
Mean [Gy]	<10Gy	10.3±1.3	8.9±0.5 (**)	10.9±1.5	9.3±0.6 (*)
V_20Gy_ [%]	<10%	11.6±3.4	10.8±2.5 (*)	14.6±3.8	12.1±2.1 (*)
**Heart**					
Mean [Gy]	<5Gy	7.7±2.4	4.0±0.7 (**)	7.9±1.4	4.6±0.6 (**)
**Spinal Cord**					
D_1%_ [Gy]	Minimise	8.8±2.5	4.3±1.2 (**)	11.4±6.5	3.7±1.4 (**)
**Esophagus**					
D_1%_ [Gy]	Minimise	10.8±2.1	6.1±2.6 (**)	16.0±7.9	6.4±3.3 (**)
**Trachea**					
D_1%_ [Gy]	Minimise	13.1±2.2	6.6±1.9 (**)	18.3±8.9	7.6±2.7 (**)

(*): p<0.05

(**): p<0.01; D_x%_: dose receive by at least X% of the volume; V_x%(Gy)_ volume receiving at least C%(Gy) of the dose.

The analysis showed that RapidPlan based optimisations lead to a systematic improvement in OAR sparing. This sparing can vary depending on the efforts put into the manual optimisation of the reference plan. As an example, the RapidPlan sparing of the heart for the left-sided patients was superior in clinics A and C compared to clinic B: the mean dose was reduced from 1.7–2.8Gy to 0.7Gy, due to the possible different optimisation strategies being more stringent in clinic B, in addition to the different contouring guidelines.

When applying the model to a new patient, the user is alerted about the structures that are flagged as outliers, i.e. presenting features different from the same parameters of the plans in the model. Evaluating the four main OARs (lung ipsi- and contra-lateral, heart, contralateral breast), for the 25 validation patients from clinic A, i.e. 100 entries, 4% were flagged as outliers out of the model range, 10% as outliers outwith the 90 percentile. For the 15 patients from clinic B (60 entries), 40% were outliers out of the range, 17% out of the 90 percentile. For the 10 patients from clinic C (40 entries), 60% were outliers out of the range, 12% out of the 90 percentile. A large portion of the outliers refer to OAR-target overlap for the ipsilateral lung. Such differences arose from the different contouring strategy adopted by clinics B and C (and not providing patients for the model configuration), allowing the inclusion of part of the lung inside the target. This fact resulted in general in a lower agreement between predicted and achieved DVH, the latter being worse for most of the cases flagged as outliers, and resulting in higher estimated mean dose to the ipsilateral lung (Table 2). A difference in the OAR-target geometry relative to the patients selected to train the model hence could lead to less accurate DVH prediction.

Regarding the difference in dose prescription of clinic C, RapidPlan allows for varying the dose prescription, using a fractionation different from that prescribed for the plans selected to train the model. This simply implies a shift in the predicted DVHs, as shown in the results presented here, pointing out the possibility to vary the fractionation scheme for the specific patient, while using the same model.

Concerning target volumes, the multi-target model developed for the SIB breast optimisation achieved all the coverage and homogeneity objectives. A general sharpening of the DVH shape for PTV_breast and PTB_boost was observed (with an increased minimum dose for clinic A and decreased maximum dose for clinic C). In particular, the model, developed from data of clinic A, succeeded also for data from clinic B, despite the different contouring strategies. In clinic B cases, the only effect was a shallower DVH for the PTV_breast due to its extension to the body surface and/or into the lungs.

The application of the model to the bilateral breast, tested to evaluate the generalisation power of the model, gave results consistent to that observed in the single breast validation. All bilateral breast cases were shown to be outliers, as expected, for the lung volume, and the geometric principal component score 1 for heart and lungs, indicating the anatomical difference between the unilateral and bilateral cases. The estimated lung DVH was different in shape with respect to the achieved result. Nevertheless the achieved DVH was generally better than the predicted one. RapidPlan based plans showed a better sparing of OARs and slightly improved PTV coverage and homogeneity. The mean dose to the heart resulted in 3.3–3.7Gy reduction on average.

Table 4 presents the results of the case-by-case pass-fail analysis conducted on all the 50 unilateral breast validation cases (corresponding to 460 pass-fail tests). Overall, RapidPlan resulted in equivalent or superior dose distributions to the reference plans in almost the totality of the cases. RapidPlan resulted in model plans being inferior to the reference in only 5 tests, i.e. in about 1.0% of the cases. Three of four “failures” occurred for PTV_breast in clinic B where the target contouring was significantly different from the model data. Concerning the bilateral breast validation experiment (180 pass-fail tests), in only 4.4% of the pass-fail tests did the reference plan result in better dose distribition than the RapidPlan. In all other cases RapidPlan based plans were superior or equivalent to the reference.

**Table 4:** Summary of the case-by-case pass-fail analysis for the selected dose-volume planning objectives for the reference and for the model-based plans for all the 50 validation cases for the single breast (25,15,10 from clinics A,B and C respectively).

	Objective	Reference fail Rapidplan pass	Reference pass RapidPlan fail	Reference fail RapidPlan fail	Reference pass RapidPlan pass
**PTV boost**					
D_1%_ [%]	<107%	0 (0%)	0 (0%)	0 (0%)	50 (100%)
V_95%_ [%]	>95%	2 (4%)	1 (2%)	3 (6%)	44 (88%)
St. Dev. [%]	<5%	2 (4%)	0 (0%)	4 (8%)	44 (88%)
**PTB breast**					
D_1%_ [%]	<PTV boost	2 (4%)	2 (4%)	5 (10%)	41 (82%)
D_95%_ [%]	>95%	18 (36%)	1 (2%)	2 (4%)	29 (58%)
**Ipsilateral Lung**					
Mean [Gy]	<10Gy	5 (10%)	0 (%)	0 (0%)	45 (90%)
V_20Gy_ [%]	<10%	5 (10%)	1 (2%)	4 (8%)	40 (80%)
**Ipsilateral Heart** (30 cases)					
Mean [Gy]	<5Gy	18 (60%)	0 (0%)	4 (13%)	8 (27%)
V_18Gy_ [%]	<5%	2 (7%)	0 (0%)	4 (13%)	24 (80%)
**Contralateral breast**					
Mean [Gy]	<3Gy	4 (8%)	0 (0%)	19 (38%)	27 (54%)

Planning time was not directly part of the study design but some qualitative data were collected. The time needed to “extract” from the patient’s database the data for model training and load them into the configuration workspace is limited to approximately 15–20 seconds per plan. To this should be added the time needed to identify the good candidates for training. The time spent to train a model is approximately 2–3 minutes. The time needed to validate a model prior to clinical use, cannot be easily assessed as this depends on the complexity of the problem and is not in any case part of a routine clinical activity. Plan optimisation with the DVH estimation engine requires approximately 15–20 seconds for the objectives generation and approximately 6–8 minutes of free-run optimisation, while the optimisation of a complex case such as breast takes 1–3 hours and depends on the complexity of the patient anatomy (in clinical practice repeated or continued optimizations are generally needed, making the planning time much longer).

## Discussion

The potential role of a knowledge-based dose-constraint prediction engine for plan optimisation of VMAT was appraised in earlier studies [8–10]. The system demonstrated the capability to generate automated and personalised dose-volume objectives leading to a plan quality that is at the same (clinically acceptable), if not better, level than the plans used to train the specific model. With the present study, devoted to whole breast irradiation, the complexity of the same problem was raised by investigating a multiple target case with SIB and rather stringent clinical dose-volume goals for some of the OARs.

A model was built using a large cohort of patients (150) from one single institute and included both left and right sided patients and validated on three groups of patients from different clinics, not used for the training. The results demonstrated a high reliability of the process. The automated and personalised constraints generated by the system, led to systematically improved dose distributions, without user interaction during the optimisation. A detailed pass-fail analysis showed that in almost the totality of the cases (99%), the RapidPlan based plans improved or matched the reference clinically approved plans. In the marginal 1% (5 data points) of the structures where RapidPlan under-performed, 3 points were observed in the breast PTV and were all from clinic B, where the contouring strategies differ substantially from that applied in the 150 training patients. Significant improved protection of the OARs was observed, particularly for the heart, with possible clinical benefit. At the same time a general gain in PTV coverage was also observed. The better results achieved with RapidPlan for clinic B and C cases (institutes not providing data for model configuration), confirm the importance of the plan selection for generating the model. The DVH prediction, and subsequent line objective determination, will refer to such a plan quality. Moreover, the placement of the line objective below the lower boundary of the prediction DVH, give strength to the knowledge, improving the average plan quality. Such an improvement is possibly larger than if the line objective would be placed along the mean prediction line. In the latter case the system would tend to preserve the same plan quality of the cases used to configure the model. Moreover, the good results of the plans generated with RapidPlan could come from the combination of the two objectives included in the model: the generated line-objective and the mean objective, both with generated priorities. The choice of the proper objectives and priorities adopted to create a model is an additional important factor related to the model quality.

A further step in the appraisal of possible advantages of the automated dose-volume objectives generation, was the model validation for bilateral breast patients. The application of VMAT to such patients [11] showed the high planning complexity of the cases that are, moreover, not frequent in routine practice. The attempt to use the model-based approach tailored to a simpler case in such a challenging situation was explored on two different groups of patients from two institutes. This RapidPlan approach showed that 96% of the pass-fail tests on the clinical dose-volume objectives were either improved or matched by the model-based plans with respect to the reference. This result also suggests that the complex bilateral breast plan process with SIB could be automated with a model based on unilateral breast cancer patients.

In summary, the model-based automation of the optimisation phase allowed one to: i) create valuable dose plans from unattended optimisation in the entire cohort of test cases; ii) realise a system that can be transparently used in multiple clinics with different contouring policies and prescription methods; iii) realise a tool that can simplify the planning activity in very challenging cases (the bilateral breast). The latter point might also suggest that the model-based automation could be beneficial in centres with limited or no direct experience in one technique, or for some types of patients; i.e. it allows a proper sharing of the planning knowledge. This was similar to the concept investigated by Good *et al* in their study [5]. Whenever the model-based approach gives results equivalent or superior to the corresponding reference plans optimised with traditional methods in the external clinic, the validity of the model and its broad-scope are proven.

The study did not address the cases of patients where the treatment of nodal stations (internal mammary or supraclavicular) should be envisaged. Dedicated studies will be devoted to this problem. On the other side, some preliminary tests were done on the use of this model for partial breast or sequential (whole breast followed by boost) irradiation. The results were all positive and this is justified by the structure of the dose-volume objectives prediction engine. In fact, within this system, each target and OAR is managed independently from the others and, from the target point of view, this opens the road to use a model built for multiple target optimisation for a simpler problem with single targets.

The study did not address, being out of scope, the issue of motion management nor the question of which respiration phase might be advisable for breast treatment. The results produced here are independent from these aspects and the methods should obviously be applied to the appropriate planning conditions.

The current study did not address many elements that could influence the plan quality, such as the dose calculation algorithm, or the arc arrangement in relation to the anatomical complexity. Those factors could modify the results, but could also compensate or enhance effects coming from the usage of the DVH prediction model. For that reason those variations were out of the scope of the present work.

## Conclusions

RapidPlan, a novel knowledge-based DVH estimation model was successfully configured, trained and validated for breast cancer. The system allowed one to improve the quality of VMAT RapidArc plans against the reference clinically accepted plans. In particular RapidPlan based plans resulted in general in superior dose distributions to reference plans for the cases provided by centres not having contributed to any case in the model training phase. Results are suggestive for the reliable application of the methodology to clinical routine.
